# Consumer Hedonic Ratings and Associated Sensory Characteristics and Emotional Responses to Fourteen Pecan Varieties Grown in Texas

**DOI:** 10.3390/plants11141814

**Published:** 2022-07-09

**Authors:** Xiaofen Du, Xinwang Wang, Adriana Muniz, Keith Kubenka

**Affiliations:** 1 Department of Nutrition and Food Sciences, Texas Woman’s University, Denton, TX 76204, USA; amuniz4@twu.edu; 2 USDA-ARS Pecan Breeding & Genetics, College Station, TX 77845, USA; xinwang.wang@usda.gov (X.W.); keith.kubenka@usda.gov (K.K.)

**Keywords:** *Carya illinoinensis*, tree nut, consumer test, pecan flavor, overall acceptance, CATA (check-all-that-apply)

## Abstract

Pecan is one of the top five most widely consumed tree nuts, and pecan nut quality is a major factor for consideration in breeding better pecan cultivars for use by producers. However, the pecan industry faces a hurdle to evaluate its nutmeat taste, and there has so far been limited evaluation of consumer attitudes toward pecan nutmeat. This study aimed to investigate the consumer (n = 198) hedonic rating, diagnostic sensory attribute intensity, and emotional response for 14 pecan samples, consisting of native/seedling and improved varieties. The results showed all kernels received positive hedonic scores (>5, 9-point hedonic scale) for overall acceptance and the acceptability of size, interior color, typical-pecan flavor, and raw-nut flavor. The primary sensory attributes (intensities > 5.0, 0–10 line scale) were typical-pecan and raw-nut flavors, followed by buttery flavor, sweetness, and astringency. Kernel off-flavors were not perceived in general. For 20 emotion-associated terms, the intensity of the satiating effect was medium, while the energizing effect was lower. The major emotional responses were healthy, satisfied, and comfort, followed by calm, interested, premium, and relaxed. Kernel variety difference was significant (*p* ≤ 0.05) for all these measured variables. Consumer overall acceptance toward pecan kernels was driven by the acceptability of flavor and interior color, flavor intensities, no off-flavors, and positive emotional responses; kernel size was not an impactful factor. The six most preferred varieties were 86TX2-1.5, Pawnee, Barton, 1997-09-0012, 1991-01-0026, and Harris Super. This study is the first to use consumer input to assess nut quality and consumption preference and will be foundational to ongoing breeding programs to develop new pecan cultivars that will better meet consumer preferences and expectations, and therefore will be accepted by the processing industry and growers.

## 1. Introduction

Pecan (*Carya illinoinensis*) is a nut tree, native to North America. The United States is the largest pecan producer and produces approximately 50% of the world’s total supply, followed by approximately 40% from Mexico [1]. Pecan has been named the official state tree in Texas, is one of Texas′s top 10 economic crops, and accounts for 16% of US production [2]. It means people from Texas are familiar with pecan commonly and may have specific feelings/emotional responses toward pecan. Pecan kernel consumption per capita per year has varied little over decades, remaining between 0.4–0.5 lbs [3]. Several specific issues exist for the US pecan industry. A minimal number of pecans are consumed outside of the Southern and Lower Plains states, even though the US is the largest pecan consumer globally and domestic utilization has exhibited an increasing trend. The pecan industry needs to efficiently market its product, prevent waste, and assure a supply of quality products at a competitive price to ensure growth, stability, and profits (personal communication with industry peers).

Over 90% of pecan nuts are processed to the edible kernel, while pecan kernels are usually eaten fresh, processed, or baked into various products, such as pies, cakes, candies, and cookies [4]. To increase the consumption of pecans and improve the competitiveness of pecan producers, an effective strategy is lacking. A few new approaches to add value to the whole pecan food chain have been identified in industry, including increased choices of raw pecan nuts, newly developed pecan co-products and by-products, and pecan shell waste reuse, diversifying the pecan product portfolio (personal communication with industry peers). Currently, the research mainly focuses on pecan tree horticultural practice and pecan kernel nutritional values [1,4,5]. Consumer acceptance would determine purchase intention and consumption, consequently determining the market demand.

So far, limited studies have investigated pecan kernel sensory quality improvement and consumer acceptance [6,7,8,9]. These studies commonly used a few terms for overall impressions, such as appearance, aroma, taste, flavor, and texture. None of these studies diagnose specific sensory attributes associated with these overall impressions. The tested products either focus on few raw pecan varieties [6], roasted kernels [7], or food products using pecans as ingredients [8,9]. Pecan’s raw kernel sensory quality is the primary driver leading to whether pecan is consumed alone or used in different food products [10]. There are more than 1000 named and documented pecan cultivars, while around 160 patented or released pecan cultivars are grown in the US [11]. Understanding consumer differentiation and attitudes towards different pecan varieties would benefit the nut industry′s variety selection, marketing, and pricing strategies.

In addition to human subjects’ sensory and hedonic perceptions of products, product-elicited emotions and the role of emotions in food choice have grown tremendously in the last decade. Product-focused emotion research provides a deeper understanding of consumers’ product experiences. Research related to emotional responses toward the consumption of pecan nuts has never been explored, although a few studies have investigated multiple emotion-related terms in tree nuts [12,13]. Of these studies, nine emotion terms were used for coated peanuts with flavorings, colorings, and peptide powder [13], and 39 emotion terms were used through a CATA (check-all-that-apply) question module for cashew nuts and coated peanut flavorings [12].

It is well documented that multiple factors impact consumer selection toward a product. One such factor is the food product itself, especially its sensory properties. On the other hand, consumer perception toward a product is influenced by demographic information, such as race, ethnicity, and region [3]. Hence, the hypothesis for the current study was that pecan kernel consumer overall liking would be determined by not only sensory attributes but also emotional responses. This study aimed to collect a panel of representative pecan nuts, including native/seedling and improved pecan cultivars, and perform affect tests, which were acceptance, associated sensory attribute intensity, and emotional responses from a group of consumers with consumption frequency in Texas.

## 2. Results

### 2.1. Pecan Kennel Consumer Hedonic Rating

As shown in Table 1 the overall liking of 14 pecan samples ranged 5.3–6.9 based on a 9-point hedonic scale, with all points above the middle point of the scale and indicating a common acceptance of these kernels. The ratings of overall liking were identified with the highest rates (6.7–6.9) for six pecan samples (86TX2-1.5, Pawnee, Barton, 1997-09-0012, 1991-01-0026, and Harris Super) and the lowest rates (5.3–5.8) for four samples (Tiemann, McMillan, 87MX4-5.5, and 1996-12-0008).

Appearance is the first sensory characteristic that consumers perceive in food, and it plays a vital role in food choice. In this study, the liking of pecan kernel size ranged from 4.6–7.4 (Table 1). Only 87MX4-5.5 was rated 4.6, while the remaining 13 kernels had ratings above 5.0. Significantly different (*p* ≤ 0.05) ratings of kernel size liking were identified within each session. Six pecan samples (Lakota, Pawnee, Barton, 1991-01-0026, 1996-12-0008, and Harris Super) were rated highest (6.7–7.4), and four pecan samples (Tiemann, 86TX2-1.5, 87MX4-5.5, and Woodside Early) were rated below 6.0. In addition to kernel size, the liking of interior color ranged from 5.3–6.9, indicating a common acceptance. Significantly different ratings of interior color liking were identified within each session as well. Five pecan samples (Pawnee, Barton, 1991-01-0026, Harris Super, and Woodside Early) were rated highest (6.5–6.9), and four pecan samples (Tiemann, McMillan, 87MX4-5.5, and 1996-12-0008) were rated below 6.0 (Table 1), indicating a light kernel color was more popular.

In addition to appearance, flavor liking has been considered the most important sensory modality [14]. The scores of the typical-pecan flavor liking for all 14 samples ranged from 5.5–6.8 (Table 1), indicating a common flavor acceptance for all tested samples. Significantly different (*p* ≤ 0.05) ratings among the seven samples within each session were identified. Overall, the results in sessions 1 and 2 showed five pecan samples (86TX2-1.5, Pawnee, Barton, 1991-01-0026, and Harris Super) were rated highest (6.5–6.9) and the other five pecan samples (Tiemann, McMillan, Williamson, 87MX4-5.5, and 1996-12-0008) were rated below 6.0. The liking for another flavor attribute, raw-nut flavor, was rated with a range of 5.3–6.7, indicating a common acceptance. A significantly different rating of raw-nut flavor among the seven samples within each session was also identified. Four pecan samples (Pawnee, Barton, 1991-01-0026, and 1997-09-0012) were rated highest (6.5–6.7), and the other five pecan samples (Tiemann, McMillan, Williamson, 87MX4-5.5, and 1996-12-0008) were rated below 6.0, indicating the majority of the improved cultivars have a positive overall liking.

It should be pointed out that the liking of four attributes (size, interior color, typical-pecan flavor, and raw-nut flavor) did not contribute equally to overall liking. Pecan kernel overall liking was significantly, positively correlated to the liking of interior color, typical-pecan flavor, and raw-nut flavor (Pearson correlation r = 0.880, 0.989, and 0.988, respectively; *p* ≤ 0.05); however, there was no significant correlation between the overall liking and the liking of kernel size.

### 2.2. Pecan Kernel Sensory Attribute Intensity

Furthermore, to evaluate the degree of liking, sensory attributes and their intensity levels were employed to diagnose the reasons for hedonic ratings. As shown in Table 2, typical-pecan flavor intensities ranged from 4.8–6.3 with the 0–10 line scale. Significantly different (*p* ≤ 0.05) ratings among the seven samples within each session were identified. Pawnee and Harris Super were the two pecan samples rated highest (above 6.0). Only one pecan (87MX4-5.5) was scored below 5.0, while all remaining 11 samples were rated between 5.0 and 6.0. Specifically, raw-nut flavor intensity ranged from 5.4–6.2, without significant differences among the seven pecan kernel samples within each session. While buttery flavor intensity ranged from 3.6–5.3, and a significant difference was identified for the seven samples within one session but no significant difference was identified for the seven samples within another session. Three kernels (86TX2-1.5, Pawnee, and Harris Super) were rated highest (5.1–5.3), while two pecan samples (87MX4-5.5 and Lakota) were rated lowest (3.6 and 4.0, respectively). The remaining nine samples were rated between 4.0 and 5.0.

The sweet taste ranged from 3.1–4.5 with significantly different (*p* ≤ 0.05) ratings among the seven samples within each session identified (Table 2). Five samples (86TX2-1.5, Pawnee, Barton, 1997-09-0012, and 1991-01-0026) were rated with 4.5, while 87MX4-5.5 was rated lowest (3.1). The ratings of sweet taste intensity for the remaining samples were in between. In addition, the astringency intensity ranged from 3.2–4.5, and significant difference was identified among the seven pecan samples within each session.

A significant correlation (*p* ≤ 0.05) was identified as correlating sensory attribute acceptability and intensities. For example, typical-pecan flavor intensity was positively correlated to typical-pecan flavor acceptability (r = 0.890), and raw-nut flavor intensity was positively correlated to raw-nut flavor acceptability (r = 0.756). A cross-modal correlation was also identified. For instance, all five attribute intensities were significantly correlated to the acceptance of typical-pecan flavor (r = 0.890, 0.738, 0.690, 0.817, −0.590 for typical-pecan flavor, raw nut flavor, buttery flavor, sweet, and astringent, respectively).

### 2.3. Pecan Kernel Off-Flavor

**Figure 1:**
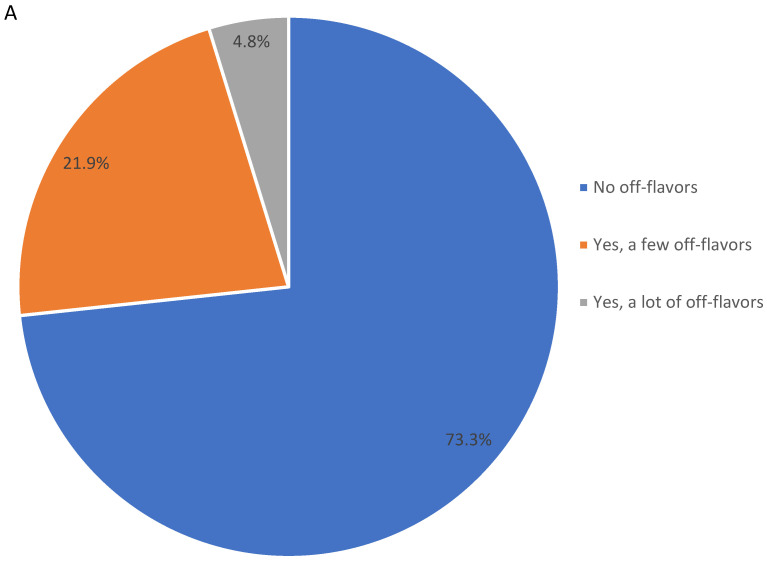

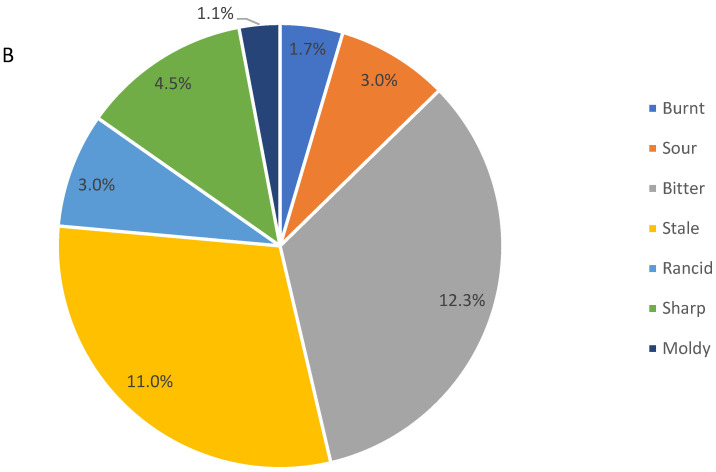
Frequencies (%) of participants (*n* = 99) indicating if they perceived any off-flavors with answers of “no”, “yes, a little”, and “yes, a lot” using a single-response question (**A**). Frequencies (%) for each off-flavor attribute calculated by dividing the total selection with the total number of panelists according to a CATA (check-all-that-apply) question (**B**).

In addition to the significant flavor attributes, consumers were asked to indicate whether they perceived any off-flavors for the pecan kernels. As shown in Figure 1A, 73.3% of participants did not perceive any off-flavors, 21.9% of participants perceived a few off-flavors, while only 4.8% perceived many off-flavors. A CATA question was followed to diagnose the significant off-flavors that participants perceived. As shown in Figure 1B, bitterness and staleness were the two major attributes associated with off-notes. The bitterness could be associated with genotypes and their ancestors or origins, while storage could also cause the perception of bitterness.

### 2.4. Consumer Emotional Response: Satiating, Energizing, and Others with CATA

**Figure 2:**
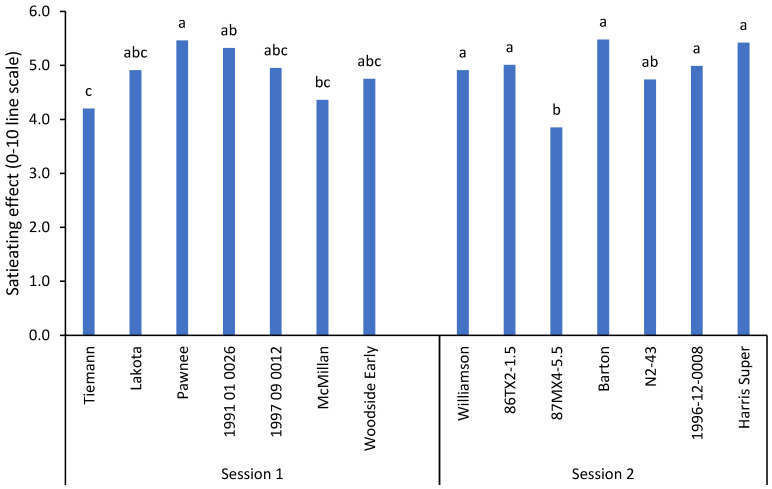
Consumer mean scores (*n* = 99) for satiating effects of 14 pecan samples on a 0–10 line scale. Different letters (a–c) within attributes indicate significant differences between samples according to one-way ANOVA and Tukey’s HSD test.

Food-evoked emotion is a crucial factor in predicting consumers’ food preferences [15]. Two emotion-related terms, satiating and energizing, were investigated for their intensity. As shown in Figure 2, the intensity of the satiating effect ranged from 3.9–5.5 on a 0–10 line scale. A significant difference (*p* ≤ 0.05) among varieties within each session was identified. Four pecan samples (Pawnee, Barton, 1991-01-0026, and Harris Super) were rated above 5.0, while 87MX4-5.5 was rated the lowest (3.9). The remaining nine samples had rates ranging from 4.0–5.0.

**Figure 3:**
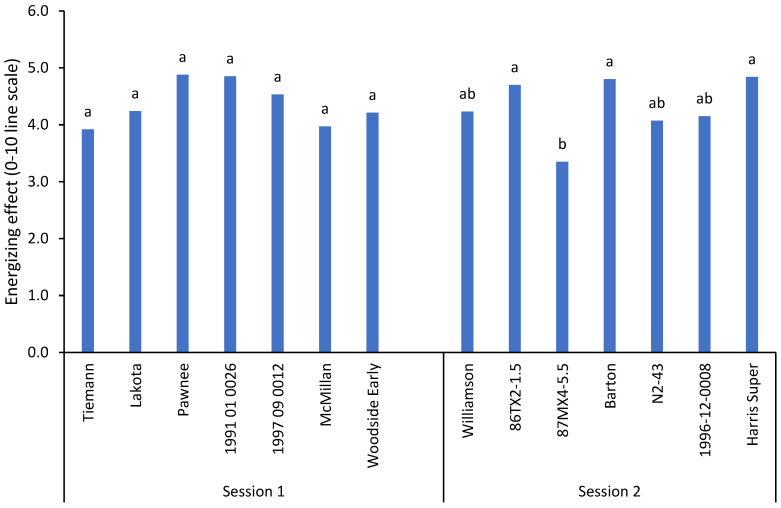
Consumer mean scores (*n* = 99) for energizing effects of 14 pecan samples on a 0–10 line scale. Different letters (a,b) within attributes indicate significant differences between samples according to one-way ANOVA and Tukey’s HSD test.

For the energizing effect, the trend for the rating was similar to that of the satiating effect. As shown in Figure 3, the intensity of energizing effect ranged from 3.4–4.9. Those four pecan samples (Pawnee, Barton, 1991-01-0026, and Harris Super) were rated highest as well (4.8 and 4.9), while Tiemann and 87MX4-5.5 were rated lowest (3.9 and 3.4, respectively). The remaining eight samples had ratings in between.

In addition, participants were asked to show their emotional responses to the pecan kernels. As shown in Table 3, the most frequently checked terms for the pecan samples were healthy (31.3–66.7%), satisfied (19.2–46.5%), and comfort (15.2–36.4%). The attributes with lower ratings were clam (11.1–25.3%), interested (10.1–24.2%), premium (9.1–31.3%), and relaxed (7.1–25.3%). The remaining responses associated with positive emotions, such as curious, homey, cheerful, and joyful, had maximum ratings below 20% for all 14 samples. The responses related to negative emotions, such as apathetic and disgusted, had low ratings with ranges of 6.1–16.2% and 4.0–12.1%, respectively. Five terms (healthy, joyful, comfort, satisfied, and disgusted) among seven varieties in session 1 and four terms (healthy, premium, joyful, and satisfied) showed a significant difference among the seven pecan samples in session 2 according to the chi-square analysis (Table 3).

Consumer emotional responses were highly associated with pecan kernel sensory properties. For example, our study showed typical-pecan flavor was significantly and positively correlated to healthy, premium, enthusiastic, interested, cheerful, joyful, comfort, satisfied, relaxed, and nostalgic, but negatively correlated to apathetic and disgusted, according to Pearson’s correlation analysis. Similarly, sweetness taste was positively correlated to healthy, premium, eager, enthusiastic, interested, cheerful, joyful, comfort, satisfied, and relaxed, but negatively correlated to apathetic, uninhibited, and disgusted.

### 2.5. Pecan Variety Difference

**Figure 4:**
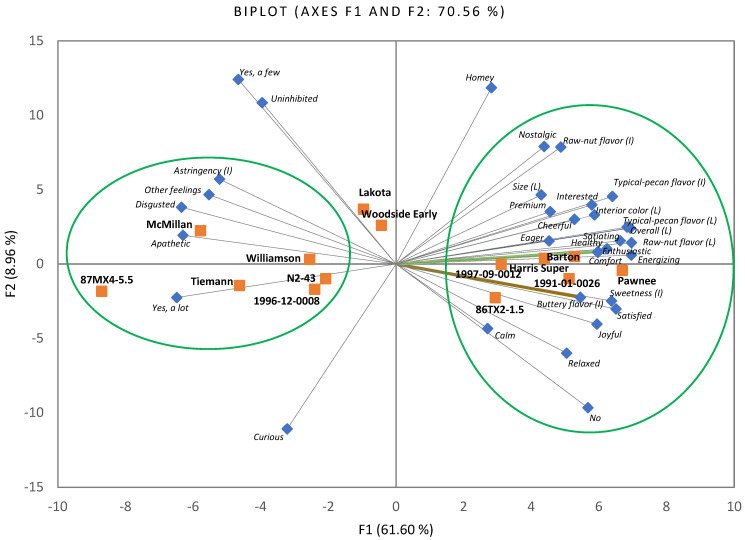
Principal component analysis (PCA) biplot of mean scores of 33 attribute ratings for 14 pecan samples. L: liking; I: intensity; No: no off-flavors; Yes, a few: yes, a few off-flavors; Yes, a lot: yes, a lot of off-flavors. According to PCA output of “Squared cosines of the variables” and “Squared cosines of the observations,” two oval circles with solid lines were created based on variables (attributes) and observations (samples) significantly separated from other samples either at positive or negative side of PC1.

According to the results of hedonic ratings, sensory attribute intensities, off-flavors, and emotional responses, the scores were dependent upon variety. A PCA was performed to visualize the underlying relationships between 33 attributes (loadings) and 14 kernel samples. The first two PCs accounted for 70.56% of the total variance, with the PC1 axis the major component (61.60%) to differentiate samples by their attributes (Figure 4).

Six samples (86TX2-1.5, Pawnee, Barton, 1997-09-0012, 1991-01-0026, and Harris Super) were separated at the positive side of PC1, possessing high scores in acceptance of all five attributes, intensities of four attributes (except astringency), no off-flavors, and 13 positive emotional responses. In contrast, the other six pecan samples (Tiemann, McMillan, Williamson, 87MX4-5.5, N2-43, and 1996-12-0008) were separated at the negative side of PC1, which were characterized with high intensities in astringency, many off-flavors, and two negative emotional responses (disgusted and apathetic). Two pecan samples (Lakota and Woodside Early), which had high ratings in emotional responses of homey and uninhabited and had a few off-flavors, were separated at the positive side of PC2. Their hedonic ratings and attribute intensity scores were in between the aforementioned ten pecan samples. The results from the PCA further confirmed the distribution of hedonic ratings, sensory attribute intensities, and emotional responses across the 14 pecan samples.

### 2.6. Drivers for Consumers’ Overall Acceptance

Among ten sensory attributes (five liking and five intensities) tested in this study, three attributes (overall acceptance, typical-pecan flavor acceptance, and typical-pecan flavor intensity) were associated with overall impressions associated with other sensory attributes. The Pearson correlation analysis indicated that the overall acceptance was significantly, positively correlated to the intensities of overall flavor, raw-nut flavor, buttery flavor, sweet taste (r = 0.900, 0.724, 0.734, 0.818, respectively), while it was significantly, negatively correlated to astringency intensity (r = −0.590) (Table 4). The overall acceptance was significantly, positively correlated to no off-flavors (r = 0.725), while negatively correlated to a few off-flavors (r = −0.571)) and a lot of off-flavors (r = −0.900). Regarding emotional responses, the overall acceptance significantly, negatively correlated to 13 terms (all positive emotion, r = 0.630–0.941) and negatively correlated to two terms (apathetic and disgusted, r =−0.850 and −0.886). Additionally, similar trends were observed for typical-pecan flavor acceptance and typical flavor intensity associated with other sensory attributes (Table 4).

In addition to the individual correlation between attributes and overall acceptance, it would be practical to investigate the drivers for consumers’ overall acceptance, which should correlate with multiple variables. To investigate the endogenous sensory attribute associated with overall acceptance, a PLS regression was performed. The PLS output showed that the first two components obtained from the analysis were used for the regression as their R^2^ and Q^2^cum values (R^2^ = 0.997, Q^2^cum = 0.979, and RMSE = 0.027) indicated a good fit model.

**Figure 5:**
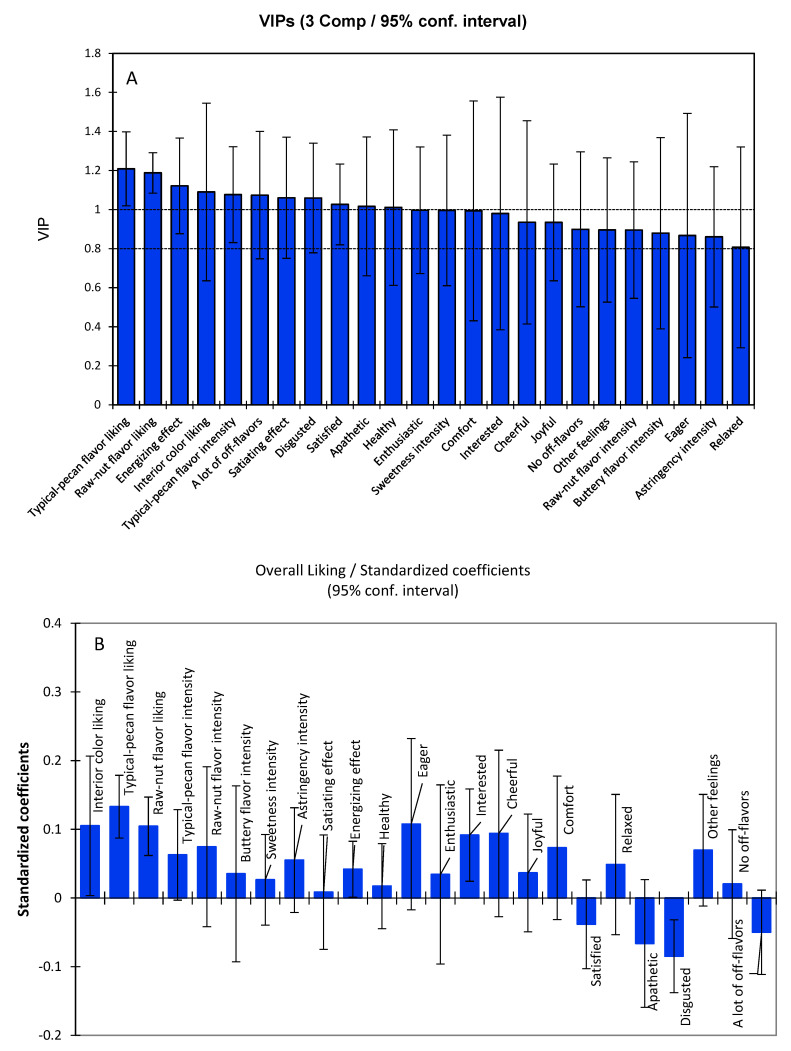
Partial least square (PLS) regression for the associations of overall acceptance and all remaining attribute likings, intensities, off-flavors, and emotional responses with variable importance in projection (VIP) > 0.8 for 14 pecan kernel samples, including (**A**) selected variables with VIPs > 0.8, and (**B**) standardized coefficients of each attribute used to predict overall acceptance. Prediction parameters were R^2^ = 0.997, Q^2^ cum = 0.979, and RMSE = 0.027, indicating a good fit model.

The overall acceptance of the 14 kernel samples was highly correlated with 24 attributes (out of 33, selected with VIP > 0.8), as shown in Figure 5A. There were 15 most impactful variables (VIP > 1.0) associated with overall acceptance, which were the acceptance of typical-pecan flavor, raw-nut flavor, and interior color; the intensities of typical-pecan flavor and sweet taste; the emotional responses of energizing, satiety, disgusted, satisfied, apathetic, healthy, enthusiastic, comfort, and interested; and many off-flavors. The results were consistent with the Pearson correlation analysis between overall acceptance and individual attributes (Table 4). Still, they were narrowed down to the most important variables by using a selection criteria of VIP > 0.1 with PLS regression analysis.

A linear regression model was built using 24 selected variables (VIP > 0.8) projected on the overall acceptance. To weigh the contributions of each attribute to the overall acceptance in the regression model, the standardized coefficients with a 95% confidence interval of PLS are displayed in Figure 5B, in which a larger coefficient indicates a more important driver. The results indicated that overall acceptance was most significantly driven by the acceptability of typical-pecan flavor, raw-nut flavor, and interior color; the intensities of raw-nut flavor; and the positive emotional response, such as eager, interested, cheerful, and comfort, while overall acceptance was driven negatively by negative emotional responses, such as disgusted and off-flavors.

## 3. Discussion

This study showed a common acceptance of the 14 pecan kernel samples according to the hedonic ratings for overall acceptance and the acceptability of appearance (size and color) and flavor (typical-pecan flavor and raw-nut flavor), although the difference in variety was significant. The findings implied that the selected pecan varieties generally had good sensory qualities. The results were consistent with a study that showed high hedonic ratings toward three pecan varieties (Kanza, Pawnee, and two natives) for the acceptance of appearance, texture, flavor, and overall [6]. The study also concluded that pecan kernel size was the major factor impacting consumer preference, which was contrary to our results. Our study indicated that kernel size had no significant impact on overall acceptance; while, hierarchically, overall acceptance was significantly, positively correlated to the acceptability of interior color, typical-pecan flavor, and raw-nut flavor. Few studies have focused on the consumer affective test for raw pecan kernels, making the results incomparable. While flavor has been commonly considered as the most impactful factor influencing consumer acceptance toward a food [14], in this study, kernel interior color was an important factor for consumer pecan acceptance as well, consistent with the fact that the color of pecan has been conventionally used as a measure of the overall kernel quality [1].

Consumer hedonic ratings are associated with the perceived sensory attributes and their intensity levels, determining the degree of pleasure experienced during consumption [16]. Descriptive sensory studies have reported the same descriptors for raw and roasted pecan kernels in that pecan nut sensory properties are characterized with 20 sensory attributes [17,18]. The evaluation of the five sensory attributes (typical-pecan flavor, raw-nut flavor, buttery flavor, sweetness, and astringency) in this study indicated that the signature flavors consumers perceived were typical-pecan flavor and raw-nut flavor, followed by buttery flavor, sweet taste, and astringency. Typical-pecan flavor, buttery flavor, sweet taste, and astringency showed a significant difference in variety, while raw-nut flavor showed consistency among the 14 pecan samples. These findings were in agreement with the conclusion in a report, in which eight sensory attributes (typical-pecan flavor, nutty-buttery, caramelized, acrid, woody, oily, astringent, and bitter) could differentiate eight pecan cultivars (Pawnee, Witte, Kanza, Major, Lakota, Giles, Maramec, and Chetopa) using a descriptive sensory analysis [18]. The flavor intensities of food depend upon the physicochemical composition, such as odor-active volatiles, taste-related non-volatiles, and textural properties. An extensive literature review yielded little information on raw pecans’ volatile and non-volatile composition [19,20]. In contrast, pecan nuts’ bioactive components and health effects have been well studied [4].

Pecans have desirable nutritional qualities, including lipids (58–66%, with a high amount of unsaturated fatty acids), carbohydrates (14%), protein (around 10%), vitamins, minerals, and phytochemicals [5,11]. As such an abundant source of unsaturated fatty acids, pecans are highly susceptible to deterioration, causing a rancid flavor [21]. This study indicated that the 14 pecan samples were overall in an excellent sensory condition, though variety difference was identified. “A lot of off-flavors” were only perceived by a small portion of participants and did not show a significant variety difference. A few studies have focused on pecan shelf-life and the results depend on multiple storage factors [1]. The in-shell pecan storage (−20 °C) for this study was only approximately two months, minimizing changes for pecan kernels.

Food taste and emotion are highly linked, and they can influence each other [22]. In this study, the satiating and energizing effects were specifically investigated using a line scale. Functional satiating foods could contribute to obesity prevention and the management of excess body weight, while obesity has been a globally prevalent health problem for the past few decades [23]. The 14 pecan varieties showed a medium level of satiating effect with significant variety differences observed, indicating a potential use of pecan kernel as a satiety food. The results reflect the different chemical compositions in pecan kernels. It has been documented that cognitive and sensory properties influence the satiating effect, and it is also dependent on the meal serving size and the macronutrients in the food [23].

An increasing number of scientific studies tend to confirm the energizing or energetic properties of foods [24,25,26]. Energizing effects can influence human behavior, induce mood changes, and affect physiological status. The energizing effect has been included in several studies for specific categorical products, such as drinks, sweet goods, and savory products [12,27,28,29,30,31,32]. No studies have focused on the energizing properties of pecan kernels. This study showed that the energizing effect was lower than the satiating effect in general with variation among the pecan varieties. The energizing effects might be associated with macronutrients, micronutrients, and sensory characterization.

Additionally, our study also included other 18 emotional responses using a CATA question. Multiple emotion-related terms have been developed in the literature. For example, the Geneva Emotion and Odor Scale (GEOS) includes 36 terms divided into six dimensions [33,34,35,36,37,38]. Of them, the EsSense Profile consists of 39 positive, negative, and unclassified terms [24]. The CD-CATA approach (consumer-defined lexicon CATA approach) ends in measuring consumers’ emotions with 39 terms [25,39]. In addition, 23 clusters of positive feelings are classified based on a self-reporting study [26]. In contrast, few studies have been published to understand consumer emotions associated with specific food products. A few studies have investigated multiple emotion-related terms in tree nuts [12,13], but not in pecan.

Of the 18 emotion-related terms, three were the most important for pecan: healthy, satisfied, and comfort, while three terms (healthy, joyful, and satisfied) showed a significant difference among the 14 pecan samples. Various sensory qualities should cause the different emotional responses to pecan kernels. Indeed, around half of these emotional responses were significantly and either positively or negatively correlated to the intensities of five attributes in this study. The relationship between sensory attributes and the emotional response has been investigated extensively in reviews [20,40], though not for tree nuts including pecan.

This consumer evaluation (hedonic, intensity, emotion) in this study was influenced by the pecan varieties. Pecan could be divided into native (or seedling) and improved varieties [11]. Native varieties are developed under natural conditions, while seeding pecan is produced from seed (the nut) and has not been bedded or grafted. Improved pecans are varieties that have been generically developed through breeding and grafting techniques. Improved pecan varieties are the dominant products (90% of the market share), while native pecan only accounts for a minimal amount of commercial share. Native varieties usually have smaller sizes and thick nutshells, making them disadvantageous for commercial purposes. Nevertheless, it has been thought that native nuts generally have a good flavor and other beneficial horticultural traits. According to this study, six samples, namely 86TX2-1.5 (native), Pawnee (improved), Barton (improved), 1997-09-0012 (breeding line), 1991-01-0026 (breeding line), and Harris Super (seedling), had the highest hedonic ratings; however, there was no universal pattern shown between the type of variety and the hedonic ratings.

In addition to pecan variety, consumer demographic features also influence food evaluation. For example, it has been found that older households, well-educated households, more wealthy households, and households without children were most likely to purchase peanuts and tree nuts [3]; moreover, the propensity to purchase nut products was different across regions, races, and ethnicities [3]. Our study was conducted in Texas, as Texas is the home of the pecan, possessing the highest cultivation and utilization of native and seedling varieties. Pecans have been integral to Texas life for centuries. The participants in our study were selected because of their high pecan nut consumption frequency, implying that these participants were more familiar with pecan and might have particular emotions toward pecan nuts.

One of the main goals of sensory and consumer research is to identify drivers of overall consumer acceptance [41]. Looking into the endogenous relationship of all measured variables in this study, the overall acceptance would be a function of several attributes and their interaction. Usually, consumers do not pay equal attention to all sensory modalities [14]. In this study, PLS regression showed that the drivers of overall acceptance were the acceptance of typical-pecan flavor and interior color, intensities of flavors, no off-flavors, and specific positive emotional responses for the pecan kernels. There are only four publications associated with consumer acceptance of pecan kernel or related pecan products [6,7,8,9], and overall acceptance is commonly included in these studies. However, no studies have focused on the driven factors for the overall acceptance of pecan kernels. This study further confirmed the importance of flavor, kernel interior color, and emotional responses. It is difficult to determine the net effect of the individual contribution of an attribute to consumer acceptance, since many attributes jointly contribute to liking and interactions exist between the attributes [42].

## 4. Materials and Methods

### 4.1. Pecan Samples

Fourteen pecan varieties, including four natives (‘Tiemann’, ‘Williamson’, 86TX2-1.5, and 87MX4-5.5), three improved (‘Barton’, ‘Lakota’, and ‘Pawnee’), three seedlings (‘Harris Super’, ‘McMillan’, and ‘Woodside Early’), and four breeding lines or crosses (N2-43, 1997-09-0012, 1991-01-0026, and 1996-12-0008), were selected for this study. These adult trees are 10–20 years old (grafted onto rootstock ‘Apache’ or ‘Riverside’, except for 86TX2-1.5 and 87TX4-5.5 on their own roots) and maintained in the repository and breeding orchards owned by USDA-ARS Pecan Breeding & Genetics Program in College Station, Texas. The information of the 14 pecan trees, including their origins, is shown in Table 5.

Approximately six pounds of nuts per variety were harvested in the fall of 2020. Nuts were collected from a single tree and all 14 collections were accomplished in multiple dates based on the nut maturation. Fresh nuts in a heavy-duty paper bag were stored in a cooling room (18 °C) to dry until all 14 samples finished (in about one week). Then all samples were shipped to Texas Woman’s University at its Denton campus in Texas. Once received, all samples were immediately stored at −20 °C until used for the consumer study within two months.

Right before the consumer tests, the pecan nuts were unshelled using a pecan nutcracker (Duke, New York, NY, USA). Each pecan nut was cracked into two halves, and the broken pieces were excluded from the consumer test. Two varieties (87TX4-5.5 and Woodside Early) had lower kernel percentages (Table 5) and could not be cracked into halves, but into quarter pieces. Photos of the nuts and kernels are shown in the Supplementary Figure S1.

### 4.2. Consumer Test—Test Design

The consumer test in this study was divided into two sessions (Table 6). Session 1 included seven varieties (Tiemann, Lakota, Pawnee, 1991-01-0026, 1997-09-0012, McMillan, and Woodside Early) and Session 2 included another set of seven varieties (Williamson, 86TX2-1.5, 87MX4-5.5, Barton, N2-43, 1996-12-0008, and Harris Super).

The test ballot included questions of five attributes for liking (size, interior color, typical-pecan flavor, raw-nut flavor, and overall acceptance) using a 9-point hedonic scale, and five attributes for intensity (typical-pecan flavor, raw-nut flavor, buttery flavor, sweet, and astringent) using a 0–10 line scale with pips, in which zero was anchored at the left end, 5 in the middle, and 10 at the right end. Typical-pecan flavor was defined as aromatics and tastes commonly associated with pecans, while raw-nut flavor was defined total nutty characteristics associated with raw pecan kernels. The ballot also included one single-choice question related to off-flavor with choices of “no”, “yes, a little”, and “yes, a lot”. A “yes, a little” or “yes, a lot” response would navigate to a CATA (check-all-that-apply) question with eight terms (burnt, sour, bitter, stale, rancid, sharp, moldy, and other off-flavors) for selection. “Sharp” meant an odor with a pungent sour impression due to oil rancidity, which produces volatile compounds. A “no” response would skip the above CATA question. The sensory attribute selection in this study was mainly determined by our preliminary study, with input from industry collaborators and the literature [18].

Two emotional response terms, satiating and energizing effects, were explored in this study using the 0–10 line scale. Satiating was defined as filling to fullness or satisfaction, while energizing was defined as giving people energy, vitality, and enthusiasm. In addition, one CATA question for emotional response, which included 18 terms with an order of healthy, premium, eager, enthusiastic, curious, interested, cheerful, joyful, comfort, satisfied, relaxed, calm, nostalgic, homey, apathetic, uninhibited, disgusted, and other feelings, was applied. The related emotional terms were selected based on the literature [25,26], and then tailored by our preliminary studies. Overall, there were 41 total measured variables in this study. More detailed information is presented in Table 6.

### 4.3. Consumer Test—Subjects

All sensory procedures were reviewed and approved by the Texas Woman’s University Institutional Review Board (IRB). Participants were recruited primarily from the senior author’s institute through bulk emails sent to students, faculty, and staff. Participants were pre-selected by excluding those who showed COVID-19 symptoms, who were allergic to nuts, and who had low pecan consumption frequency using Google Form. The consumption frequencies included in the selection were at least once per week, a few times per month, once per month, and at least several times a year. The individuals with no previous consumption were excluded from the test (or participation). A total of 173 voluntary participants were recruited, with 99 participants for each study. There were 25 participants attending both sessions. Eligible participants were notified via emails and scheduled to participate in the test. A panel of 99 participants in Session 1 consisted of 15 males and 84 females, and their ages ranged 18 or older, with the majority of participants (68%) identifying in the 18–25 age group followed by 19% in the 26–35 age group. Another panel of 99 participants in Session 2 consisted of 20 males and 79 females. Their ages ranged from 18 or older, with most participants (68%) identifying in the 18–25 age group followed by 17% in the 26–35 age group. Therefore, the demographic profile for the two sessions was very similar.

### 4.4. Consumer Test—Test Procedure

The test procedure was standardized in the senior author’s lab [43]. Upon arriving at the sensory lab, participants were signed-in and read, signed, and dated the consent form. Then a trained researcher escorted participants into the partitioned sensory booth illuminated with incandescent lighting and discussed the sensory booth setup and testing procedures. Participants were encouraged to ask questions before starting any sample evaluation.

Participants received a tray containing seven plastic taste cups (each cup contained five kernel halves representing each sample), one 354-mL cup of drinking water, and one piece of napkin. The cup was labelled with a randomized three-digit code and randomized using randomized, balanced blocks (Williams Latin squares design). Each participant received one iPad installed with the Compusense Cloud version 19 software (Compusense, Guelph, ON, Canada).

The electronic, written test ballot included the instructions and a score sheet. Participants were instructed to rinse their mouth with water first, taste one pecan sample at a time, and swish their mouth with water before sampling the next kernel. There was a 25 sec break between each pecan sample within the same session. Although the participants were encouraged to take their time, each panelist’s entire sensory test session was approximately 30 min. After evaluation, participants completed an exit survey, including six demographic questions about age, gender, pecan consumption frequency, type of pecan-related food consumption, other nut consumption, and purchase intent. Each participant received a cash honorarium for participation.

### 4.5. Statistical Analysis

Data in Sessions 1 and 2 were treated as two separate population samples, although each separate session of 99 participants should represent the same population according to our participant recruitment pool and data pre-check. A one-way analysis of variance (ANOVA) was used to examine the variation in consumer hedonic ratings, flavor intensities, and satiating and energizing effects among the 14 pecan kernel samples. These evaluated attributes were “dependent variables”, while pecan variety was a “factor” in SPSS software. Tukey’s honestly significant difference (HSD) test for pairwise comparisons was performed for the pecan kernel samples. A chi-square analysis was conducted for frequency data for off-flavors with responses of “no”, “yes, a little”, and “yes, a lot” (single-response question), as well as for emotional response with CATA (18 terms). ANOVA, HSD, and Chi-square analyses were applied with SPSS version 25 (IBM SPSS Statistics, Armonk, NY, USA).

A principal components analysis (PCA) assessed the similarities and differences among the 14 pecan kernel samples using the covariance matrix with attribute liking, intensities, off-flavors, and emotional responses as loading-values. Pearson correlation coefficients were calculated to determine the relationship between all above measured variables and three overall impressions (overall liking, typical-pecan flavor liking, and typical-pecan flavor intensity). A partial least square (PLS) regression was conducted to identify attributes projected to the overall acceptance of pecan kernel samples. A selection criterion of variable importance in projection (VIP) > 0.8 was performed to select those variables significantly contributing to overall acceptance. PCA, Pearson correlation, and PLS were performed using XLSTAT 2019 (Addinsoft, New York, NY, USA). A statistical significance was achieved with *p* ≤ 0.05.

## 5. Conclusions

The research provided insight into consumer acceptance toward 14 selected pecan varieties. The kernel received positive hedonic scores for overall acceptance and attribute acceptability, indicating that consumers generally liked the 14 pecan varieties. Sensory intensity evaluation showed the primary attributes were typical-pecan flavor and raw-nut flavor, which provided insight into whether it was a product that people liked or disliked, along with off-flavors. In addition, emotional responses added values for a further understanding of the consumer satisfaction consumption of pecan kernels. Overall, the measured variables in this study showed variety differences, with the improved varieties being overall better than the native and seedling varieties. The driven factors for consumers’ overall acceptance could be considered by pecan breeders for the development of superior pecan cultivars in the breeding program. However, consumer acceptance toward pecan kernels was uniformly driven by flavor acceptance, flavor intensity, no off-flavors, and positive emotional responses. The appeal of the kernel flavor is the most impactful factor driving consumer consumption. The data obtained will be a guide for breeders to develop new pecan types that will be accepted by producers for incorporation into their commercial orchards. The potential limitation for the current study is that the results did not necessarily represent the global population, but just consumers in Texas.

## Figures and Tables

**Table 1:** Mean scores of five liking attributes for the consumer tests of 14 pecan varieties.

	Size Liking	Interior Color Liking	Typical-Pecan Flavor Liking	Raw-Nut Flavor Liking	Overall Liking
Session 1					
Tiemann	5.9 ^bc^	5.7 ^bc^	5.6 ^c^	5.6 ^bc^	5.6 ^c^
Lakota	7.0 ^a^	6.3 ^ab^	6.4 ^a^	6.2 ^ab^	6.4 ^ab^
Pawnee	7.4 ^a^	6.8 ^a^	6.8 ^a^	6.7 ^a^	6.9 ^a^
1991-01-0026	7.0 ^a^	6.9 ^a^	6.8 ^a^	6.7 ^a^	6.9 ^a^
1997-09-0012	6.1 ^b^	6.3 ^ab^	6.5 ^a^	6.4 ^a^	6.7 ^a^
McMillan	6.1 ^b^	5.4 ^c^	5.7 ^bc^	5.5 ^c^	5.6 ^bc^
Woodside Early	5.3 ^c^	6.5 ^a^	6.4 ^ab^	6.3 ^ab^	6.5 ^a^
*Significance*	***	***	***	***	***
Session 2					
Williamson	6.5 ^bc^	6.0 ^bc^	5.8 ^c^	5.8 ^bc^	6.0 ^cd^
86TX2-1.5	5.2 ^d^	6.1 ^abc^	6.7 ^a^	6.4 ^ab^	6.7 ^ab^
87MX4-5.5	4.6 ^d^	5.7 ^cd^	5.5 ^c^	5.3 ^c^	5.3 ^d^
Barton	7.3 ^a^	6.5 ^ab^	6.8 ^a^	6.5 ^a^	6.8 ^a^
N2-43	6.0 ^c^	6.2 ^abc^	6.0 ^bc^	6.0 ^abc^	6.1 ^bc^
1996-12-0008	6.9 ^ab^	5.3 ^d^	5.9 ^c^	5.8 ^abc^	5.8 ^cd^
Harris Super	6.7 ^ab^	6.7 ^a^	6.7 ^ab^	6.5 ^a^	6.8 ^ab^
*Significance*	***	***	***	***	***

Different letters (a–d) within each attribute across different pecan samples per session indicate significant difference between samples according to one-way ANOVA and Tukey’s HSD test (*** *p* < 0.001).

**Table 2:** Mean scores of five intensity attributes for the consumer tests of 14 pecan varieties.

	Typical-Pecan Flavor intensity	Raw-Nut Flavor Intensity	Buttery Flavor Intensity	Sweetness Intensity	Astringency Intensity
Session 1					
Tiemann	5.3 ^b^	5.4 ^a^	4.5 ^a^	3.8 ^ab^	3.7 ^ab^
Lakota	5.8 ^ab^	6.1 ^a^	4.0 ^a^	3.7 ^ab^	4.3 ^a^
Pawnee	6.2 ^a^	6.2 ^a^	5.1 ^a^	4.5 ^a^	3.2 ^b^
1991-01-0026	5.9 ^ab^	5.9 ^a^	4.9 ^a^	4.5 ^a^	3.3 ^b^
1997-09-0012	5.8 ^ab^	5.7 ^a^	4.7 ^a^	4.5 ^a^	3.5 ^b^
McMillan	5.1 ^b^	5.4 ^a^	4.6 ^a^	3.8 ^ab^	3.8 ^ab^
Woodside Early	5.9 ^ab^	5.9 ^a^	4.7 ^a^	3.9 ^ab^	4.1 ^ab^
*Significance*	***	ns	ns	**	*
Session 2					
Williamson	5.5 ^abc^	5.5 ^a^	4.5 ^abc^	3.8 ^ab^	4.2 ^ab^
86TX2-1.5	5.8 ^ab^	5.6 ^a^	5.3 ^a^	4.5 ^a^	3.6 ^ab^
87MX4-5.5	4.8 ^c^	5.2 ^a^	3.6 ^c^	3.1 ^b^	4.5 ^a^
Barton	5.9 ^ab^	5.5 ^a^	4.8 ^ab^	4.5 ^a^	3.4 ^b^
N2-43	5.1 ^bc^	5.2 ^a^	4.7 ^ab^	3.7 ^ab^	4.0 ^ab^
1996-12-0008	5.4 ^bc^	5.5 ^a^	4.2 ^bc^	4.2 ^a^	3.6 ^ab^
Harris Super	6.3 ^a^	6.1 ^a^	5.3 ^a^	4.1 ^a^	3.6 ^ab^
*Significance*	***	ns	***	***	**

Different letters (a–c) within each attribute across different pecan samples per session indicate significant difference between samples according to one-way ANOVA and Tukey’s HSD test (*, **, *** *p* < 0.05, *p* < 0.01, and *p* < 0.001, respectively). “ns”: no significance.

**Table 3:** Frequency (ticked responses, %) of each emotional term for 14 pecan samples using a CATA (check-all-that-apply) question module.

	Healthy	Premium	Eager	Enthusiastic	Curious	Interested	Cheerful	Joyful	Comfort	Satisfied	Relaxed	Calm	Nostalgic	Homey	Apathetic	Uninhibited	Disgusted	Others
Session 1																		
Tiemann	37.4 ^abc^	10.1 ^a^	4.0 ^a^	8.1 ^a^	18.2 ^a^	18.2 ^a^	9.1 ^a^	7.1 ^a^	15.2 ^c^	26.3 ^ab^	16.2 ^a^	24.2 ^a^	8.1 ^a^	10.1 ^a^	16.2 ^a^	12.1 ^a^	10.1 ^ab^	3.0 ^a^
Lakota	50.5 ^abc^	18.2 ^a^	9.1 ^a^	10.1 ^a^	12.1 ^a^	21.2 ^a^	10.1 ^a^	6.1 ^a^	21.2 ^abc^	26.3 ^ab^	12.1 ^a^	16.2 ^a^	15.2 ^a^	15.2 ^a^	13.1 ^a^	10.1 ^a^	9.1 ^ab^	4.0 ^a^
Pawnee	66.7 ^c^	20.2 ^a^	7.1 ^a^	15.2 ^a^	11.1 ^a^	24.2 ^a^	10.1 ^a^	14.1 ^a^	36.4 ^b^	46.5 ^a^	25.3 a	21.2 ^a^	11.1 ^a^	18.2 ^a^	6.1 ^a^	7.1 ^a^	4.0 ^ab^	0.0 ^a^
1991-01-0026	50.5 ^abc^	15.2 ^a^	11.1 ^a^	14.1 ^a^	12.1 ^a^	24.2 ^a^	10.1 ^a^	11.1 ^a^	33.3 ^abc^	43.4 ^a^	15.2 ^a^	25.3 ^a^	8.1 ^a^	14.1 ^a^	10.1 ^a^	4.0 ^a^	1.0 ^a^	0.0 ^a^
1997-09-0012	52.5 ^abc^	18.2 ^a^	10.1 ^a^	9.1 ^a^	9.1 ^a^	19.2 ^a^	16.2 ^a^	17.2 ^a^	28.3 ^abc^	39.4 ^a^	18.2 ^a^	20.2 ^a^	11.1 ^a^	18.2 ^a^	9.1 ^a^	6.1 ^a^	4.0 ^ab^	2.0 ^a^
McMillan	31.3 ^ac^	15.2 ^a^	7.1 ^a^	5.1 ^a^	11.1 ^a^	19.2 ^a^	7.1 ^a^	6.1 ^a^	15.2 ^ac^	19.2 ^b^	10.1 ^a^	11.1 ^a^	8.1 ^a^	19.2 ^a^	14.1 ^a^	13.1 ^a^	12.1 ^b^	6.1 ^a^
Woodside Early	50.5 ^abc^	13.1 ^a^	6.1 ^a^	8.1 ^a^	8.1 ^a^	21.2 ^a^	8.1 ^a^	6.1 ^a^	25.3 ^abc^	30.3 ^ab^	13.1 ^a^	16.2 ^a^	10.1 ^a^	20.2 ^a^	10.1 ^a^	14.1 ^a^	6.1 ^ab^	4.0 ^a^
*Significance*	***	ns	ns	ns	ns	ns	ns	*	**	***	ns	ns	ns	ns	ns	ns	*	ns
Session 2																		
Williamson	49.0 ^ab^	24.0 ^abc^	8.3 ^a^	11.5 ^a^	15.6 ^a^	18.8 ^a^	8.3 ^a^	6.3 ^b^	20.8 ^a^	34.4 ^ab^	6.3 ^a^	11.5 ^a^	6.3 ^a^	8.3 ^a^	13.5 ^a^	8.3 ^a^	9.4 ^a^	4.2 ^a^
86TX2-1.5	48.4 ^ab^	23.2 ^abc^	8.4 ^a^	11.6 ^a^	17.9 ^a^	24.2 ^a^	12.6 ^a^	14.7 ^ab^	21.1 ^a^	31.6 ^ab^	21.1 ^a^	17.9 ^a^	9.5 ^a^	12.6 ^a^	6.3 ^a^	4.2 ^a^	4.2 ^a^	4.2 ^a^
87MX4-5.5	36.8 ^b^	9.5 ^ac^	6.3 ^a^	5.3 ^a^	16.8 ^a^	10.5 ^a^	3.2 ^a^	3.2 ^b^	21.1 ^a^	20.0 ^a^	11.6 ^a^	13.7 ^a^	5.3 ^a^	8.4 ^a^	15.8 ^a^	8.4 ^a^	11.6 ^a^	4.2 ^a^
Barton	56.3 ^ab^	31.3 ^b^	10.4 ^a^	12.5 ^a^	7.3 ^a^	24.0 ^a^	15.6 ^a^	17.7 ^a^	31.3 ^a^	40.6 ^b^	17.7 ^a^	11.5 ^a^	13.5 ^a^	12.5 ^a^	6.3 ^a^	8.3 ^a^	5.2 ^a^	2.1 ^a^
N2-43	44.8 ^ab^	14.6 ^abc^	8.3 ^a^	6.3 ^a^	12.5 ^a^	19.8 ^a^	9.4 ^a^	12.5 ^ab^	18.8 ^a^	26.0 ^ab^	13.5 ^a^	13.5 ^a^	8.3 ^a^	10.4 ^a^	12.5 ^a^	10.4 ^a^	5.2 ^a^	5.2 ^a^
1996-12-0008	46.9 ^ab^	11.5 ^c^	7.3 ^a^	4.2 ^a^	17.7 ^a^	18.8 ^a^	3.1 ^a^	8.3 ^ab^	15.6 ^a^	34.4 ^ab^	13.5 ^a^	11.5 ^a^	10.4 ^a^	9.4 ^a^	9.4 ^a^	7.3 ^a^	8.3 ^a^	2.1 ^a^
Harris Super	62.1 ^a^	20.0 ^abc^	8.4 ^a^	12.6 ^a^	15.8 ^a^	20.0 ^a^	14.7 ^a^	17.9 ^a^	26.3 ^a^	40.0 ^ab^	15.8 ^a^	12.6 ^a^	11.6 ^a^	14.7 ^a^	6.3 ^a^	7.4 ^a^	4.2 ^a^	2.1 ^a^
*Significance*	*	**	ns	ns	ns	ns	ns	**	ns	*	ns	ns	ns	ns	ns	ns	ns	ns

The frequency was calculated by dividing the sum of response for each term with the total number of participants (*n* = 99). Different letters within attributes across different pecan samples indicate significant differences between samples according to Chi-square analysis with *p* ≤ 0.05. “ns”: no significance. *, **, and ***: significant at *p* < 0.05, *p* < 0.01, and *p* < 0.001, respectively.

**Table 4:** Pearson correlation coefficient (*r*) between overall impressions (overall acceptance, typical-pecan flavor liking, and intensity) and all 33 variables in a significant level of *p* ≤ 0.05.

Variables	Overall Liking	Typical-Pecan Flavor Liking	Typical-Pecan Flavor Intensity
Size liking	0.499	0.500	**0.557**
Interior color liking	**0.880**	**0.839**	**0.782**
Typical-pecan flavor liking	**0.989**	**1**	**0.890**
Raw-nut flavor liking	**0.988**	**0.979**	**0.907**
Overall liking	**1**	**0.989**	**0.900**
Typical-pecan flavor intensity	**0.900**	**0.890**	**1**
Raw-nut flavor intensity	**0.724**	**0.738**	**0.878**
Buttery flavor intensity	**0.734**	**0.690**	**0.703**
Sweetness intensity	**0.818**	**0.817**	**0.752**
Astringency intensity	**−0.590**	**−0.590**	**−0.543**
Satiating effect	**0.881**	**0.867**	**0.858**
Energizing effect	**0.941**	**0.927**	**0.899**
Healthy	**0.842**	**0.815**	**0.871**
Premium	**0.636**	**0.619**	**0.533**
Eager	**0.680**	**0.694**	0.409
Enthusiastic	**0.830**	**0.803**	**0.806**
Curious	**−0.533**	−0.509	−0.363
Interested	**0.814**	**0.809**	**0.752**
Cheerful	**0.764**	**0.728**	**0.657**
Joyful	**0.778**	**0.758**	**0.643**
Comfort	**0.831**	**0.816**	**0.711**
Satisfied	**0.817**	**0.786**	**0.795**
Relaxed	**0.640**	**0.652**	**0.588**
Calm	0.372	0.367	0.330
Nostalgic	**0.630**	**0.679**	**0.709**
Homey	0.474	0.469	0.526
Apathetic	**−0.850**	**−0.854**	**−0.800**
Uninhibited	−0.492	−0.517	−0.336
Disgusted	**−0.886**	**−0.856**	**−0.707**
Other feelings	**−0.658**	**−0.655**	**−0.682**
No off-flavors	**0.725**	**0.702**	**0.574**
Yes, a little off-flavors	**−0.571**	**−0.553**	−0.401
Yes, a lot off-flavors	**−0.900**	**−0.869**	**−0.844**

Numbers in bold indicate significant correlations (*p* ≤ 0.05).

**Table 5:** Pecan sample information, including tree origination and background information.

Plant ID	Type *	Origin
Tiemann	Native	Colorado river bottom near La Grange in Fayette County, TX.
Williamson	Native	Mill Creek in Johnson County, OK in 1911 by E.W. Kirkpatrick.
86TX2-1.5	Native	Zavala County, TX
87MX4-5.5	Native	Hidalgo, MX
Barton	Improved	A progeny of ‘Mahan’ x ‘Major’ cultivars, made by L. D. Romberg at Brownwood, TX in 1964. USDA released in 2007.
Lakota	Improved	A progeny of ‘Mohawk’ x ‘Starking Hardy Giant’, made by L. D. Romberg, Brownwood, TX in 1963. USDA released in 1984.
Pawnee	Improved	A progeny of ′Moore’ x ‘Success’, made by L. D. Romberg in Brownwood, TX in 1937. USDA released in 1953.
N2-43	Cross	A progeny of ′Nuggett′ x ′Western′ originated In NM and was grafted onto ‘Riverside’ rootstock in Brownwood, TX in 1966.
1991-01-0026	Cross	A progeny from ′Barton′ x ′Pawnee′, made in Brownwood, TX by T. E. Thompson in 1991.
1996-12-0008	Cross	A progeny from ′Barton′ x (′Cheyenne′ x ′Pawnee′), made in Brownwood, TX by T. E. Thompson in 1996.
1997-09-0012	Cross	A progeny from ′Osage′ x (′Cheyenne′ x ′Pawnee′), made in Brownwood, TX by T. E.Thompson in 1997.
Harris Super	Seedling	A chance seedling found at Gunnison in Bolivar county, MS in 1952.
McMillan	Seedling	Holly Hills in Baldwin County, AL.
Woodside Early	Seedling	Originated near Alexandria in Rapides Parish, LA.

* Crosses are breeding lines but not released for commercial use. Improved varieties are cross but released.

**Table 6:** Pecan kernel consumer test design.

Session	Sample	Liking and Scale	Intensity and Scale	Off-Flavor	Feeling
Session 1 Participants (N = 99)	Tiemann	Five hedonic questions: *Size* *Interior color* *Typical-pecan flavor* *Raw-nut flavor* *Overall*	Seven intensity questions: *Typical-pecan flavor Raw-nut flavor* *Buttery flavor* *Sweetness* *Astringency* *Satiating* *Energizing*	Single response question: *No* *Yes, a few* *Yes, a lot*	A CATA question related to feeling: *Healthy* *Premium* *Eager* *Enthusiastic* *Curious* *Interested* *Cheerful* *Joyful* *Comfort* *Satisfied* *Relaxed* *Calm* *Nostalgic* *Homey* *Apathetic* *Uninhibited* *Disgusted* *Other feelings*
	Lakota				
	Pawnee				
	1991-01-0026				
	1997-09-0012 McMillan				
	Woodside Early				
Session 2 Participants (N = 99)	Williamson	9-point hedonic scale: *1 = dislike extremely* *2 = dislike very much* *3 = dislike moderately* *4 = dislike slightly* *5 = either like or dislike* *6 = like slightly* *7 = like moderately* *8 = like very much* *9 = like extremely*	0–10 line scale: *Pips* *0 = none* *5 = moderate* *10 = extremely strong*	If yes, navigate to a multiple choice question for off-flavor: *Burnt* *Sour* *Bitter* *Stale* *Rancid* *Sharp* *Moldy* *Other off-flavors*	
	86TX2-1.5				
	87MX4-5.5				
	Barton				
	N2-43				
	1996-12-0008				
	Harris Super				

CATA: check-all-that-apply.

## Data Availability

Our data are available for review.

## Supplementary Materials

Supporting information can be downloaded at: https://www.mdpi.com/article/10.3390/plants11141814/s1, Figure S1: Photos of nuts and kernels used for the consumer test in this study.
