# Supplementary figures and images for: Consumer Hedonic Ratings and Associated Sensory Characteristics and Emotional Responses to Fourteen Pecan Varieties Grown in Texas
Source: Plants (Basel). 2022 Jul 9;11(14):1814. doi: 10.3390/plants11141814 (PMC9318619; doi:10.3390/plants11141814)

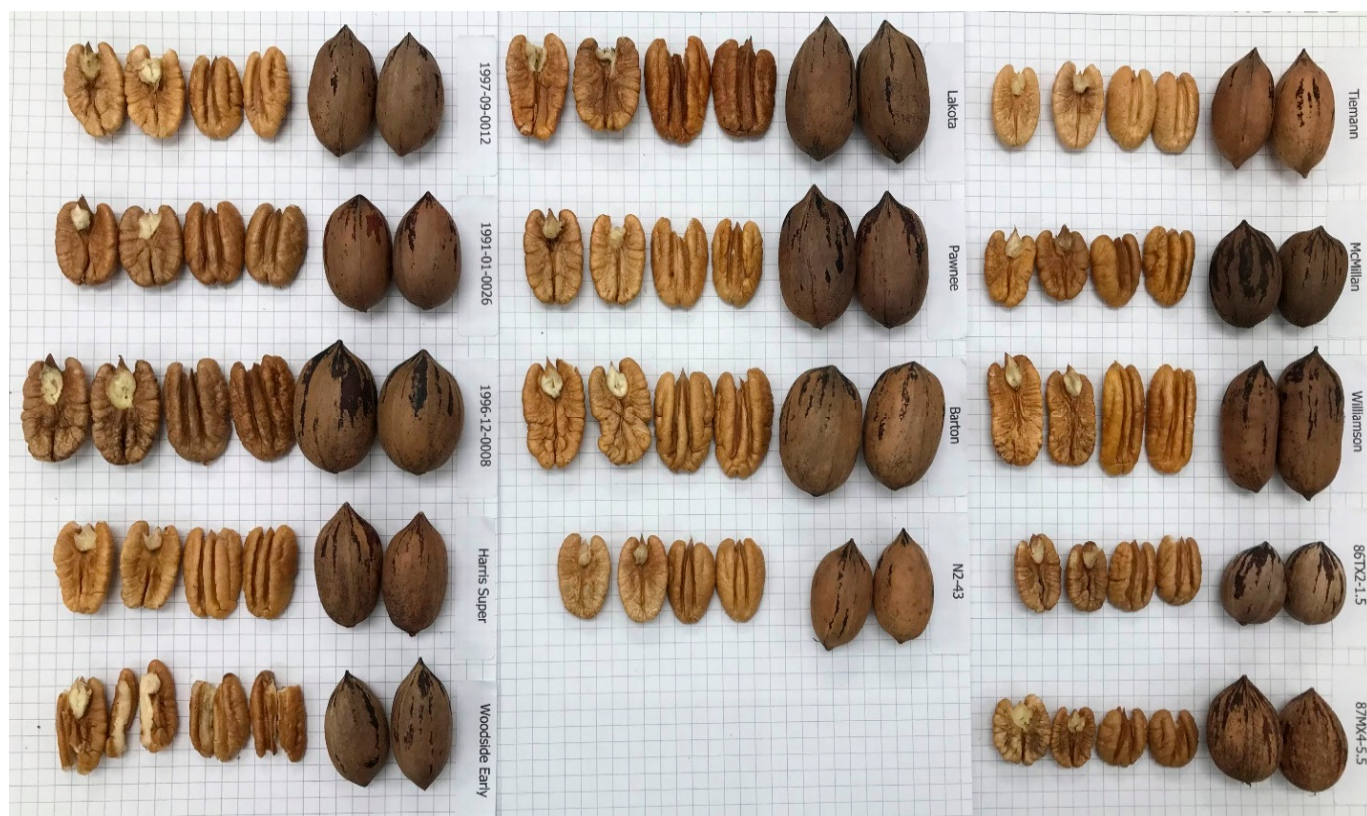

**Figure S1.** Photos of nuts and kernels used for the consumer test in this study.

Supplement: Supplementary file 1 [file plants-11-01814-s001.zip › plants-1776705-supplementary.pdf]
